# Effects of Dexmedetomidine on Systemic Inflammation and Postoperative Complications in Laparoscopic Pancreaticoduodenectomy: A Double-blind Randomized Controlled Trial

**DOI:** 10.1007/s00268-022-06802-8

**Published:** 2022-11-05

**Authors:** Yan-Xin Chen, Lin Du, Li-Nan Wang, Yong-Yong Shi, Min Liao, Min Zhong, Gao-Feng Zhao

**Affiliations:** Department of Anaesthesiology, The Second Affiliated Hospital of Guangzhou University of Chinese Medicine, Guangzhou, Guangdong PR China

## Abstract

**Background:**

Laparoscopic pancreaticoduodenectomy (LPD) may induce intense inflammatory response which might be related to the patient’s outcomes. Clinical dexmedetomidine (DEX) has been widely used for opioid-sparing anesthesia and satisfactory sedation. The objective of this study was to investigate the influence of DEX on inflammatory response and postoperative complications in LPD.

**Methods:**

Ninety-nine patients undergoing LPD were randomly assigned to two groups: normal saline (NS) and DEX. The primary outcome was the neutrophil-to-lymphocyte ratio (NLR) differences postoperatively within 48 h. Secondary outcomes were postoperative complications, the length of postoperative hospital stay and the incidence of ICU admission. Other outcomes included anesthetics consumption and intraoperative vital signs.

**Results:**

NLR at postoperative day 2 to baseline ratio decreased significantly in the DEX group (*P* = 0.032). Less major complications were observed in the DEX group such as pancreatic fistula, delayed gastric emptying and intra-abdominal infection (NS vs. DEX, 21.7% vs. 13.6%, *P* = 0.315; 10.9% vs. 2.3%, *P* = 0.226; 17.4% vs. 11.4%, *P* = 0.416, respectively) though there were no statistical differences. Three patients were transferred to the ICU after surgery in the NS group, while there was none in the DEX group (*P* = 0.242). The median postoperative hospital stay between groups were similar (*P* = 0.313). Both intraoperative propofol and opioids were less in the DEX group (*P* < 0.05).

**Conclusions:**

Intraoperative DEX reduced the early postoperative inflammatory response in LPD. It also reduced the use of narcotics that may related to reduced major complications, which need additional research further.

## Introduction

Pancreatic cancer is currently the seventh leading cause of cancer death worldwide and the third leading cause of cancer-related death in the USA [1]. Surgical manipulation is the most effective treatment for these tumors such as carcinoma of pancreatic head, ampulla carcinoma and cholangiocarcinoma [2]. Laparoscopic pancreatoduodenectomy (LPD) is one of the most challenging abdominal operations for pancreatic and periampullary tumors, which involves complex intra-abdominal dissection and reconstruction techniques that may impair patient’s immune function [3, 4]. Growing evidence indicates that immune state and inflammatory responses play a pivotal role in the development of clinically relevant postoperative complications after pancreaticoduodenectomy [5]. A variety of immune cells play their respective roles in the process of inflammation and tumors. The neutrophil-to-lymphocyte ratio (NLR) is usually served as potential surrogate markers of systemic inflammations [6]. It is an inexpensive, widely available parameters that could be used as an indicator to predict the risk of recurrence [7], and prognosis of many types of cancers [8]. Anesthetic agents in the perioperative period could also exert their unique immune and inflammatory regulatory effects and affect the outcomes of surgery [9]. Reasonable anesthesia selection can effectively reduce the level of inflammation and improve prognosis.

Dexmedetomidine (DEX) is a highly selective *α*2 receptor agonist and has broad pharmacologic effects such as anesthesia, analgesia, sedation, and anxiolysis [10]. It is widely used in the perioperative period and has been widely accepted as an adjunct to general anesthesia, associated with opioid-sparing and organ protective effection. In general, it can attenuate the surgical stress response by inhibiting the hypothalamic–pituitary–adrenal (HPA) axis and the sympathetic-adrenal-medullary (SAM) axis, reducing the release of catecholamines and cortisol. Besides, both in vivo and in vitro studies have confirmed that DEX can reduce the levels of inflammatory factors in the circulation, thereby posing an anti-inflammatory shift [11−14]. A single-center, retrospective, cohort study involving 2452 consecutive patients who underwent cardiac surgery showed that perioperative DEX infusion significantly reduced the mortality of postoperative 5-year. Another clinical trial showed that the elderly patients in ICU impacting of DEX increase the 2-year survival rate postoperatively [15]. The application of DEX has also been implied to influence oncological prognosis [16]. Despite the possible beneficial effects of DEX on perioperative patients, its role in pancreatic cancer surgery has not been well established.

As a result of the potential immunomodulation of DEX, we hypothesized that the use of DEX could attenuate the inflammatory response induced by LPD. In parallel, we also investigated the incidence of postoperative complications related.

## Methods

### Ethics

Ethical approval for this study (Z2017-129–01) was provided by the Ethics Committee of Guangdong Provincal Hospital of Chinese Medicine (Chairperson Professor Jun Liu), Guangzhou, China, on 8 September 2017. This randomized, double-blinded, controlled trial was registered on Chinese Clinical Trial Registry (ChiCTR 1,800,017,065, July 10, 2018) and conducted between 10 July 2018 and 10 January 2022 at the Department of Anaesthesiology of The Second Affiliated Hospital of Guangzhou University of Chinese Medicine, China. Written informed consent was obtained from each eligible patient on the day before surgery.

### Study design

The inclusion criteria for patients were that they planned to receive LPD, aged 18 to 65 years, body mass index (BMI) <28.0 kg m^−2^ and American Society of anesthesiologists (ASA) classification: Grade I-III. The exclusion criteria were as follows: severe heart, lung, hepatic or renal diseases; mental disorder; substance abuse; long-term use of sedative hypnotics, antidepressants or other psychotropics; and long-term hormone used and sinus bradycardia.

### Anesthesia management and groups

To ensure the randomization of groups, random number was generated by computer and stored in sequentially numbered envelopes. A nurse who did not participate in the trial prepared drug X [DEX or normal saline (NS)] according to the number in the envelope. Only this nurse was aware of patient allocation. General anaesthesia was induced with propofol (TCI 3ug ml^−1^) and drug X 3 µg kg^−1^ h^−1^ infusion for 10 min. Tracheal intubation was faciliated by sufentanil (0.3ug kg^−1^) and cis-atracurium (0.15 mg kg^−1^), and then anesthesia was maintained with propofol, cis-atracurium, remifentanil infusion and 1% sevoflurane in oxygen (FiO_2_ = 0.6). Drug X was continuous infused with 0.3 µg kg^−1^ h^−1^ until the surgical specimen resected. Depth of anesthesia was monitored by Nacrotrend-Compact (MT MonitorTechnik GmbH&Co.KG, D-24576 Bad Bramstect) and the index was maintained between 37 and 64. A volume-controlled ventilation mode with a tidal volume of 8 ml kg^−1^ was performed for eligible patients, and the total flow rate of 2 L·min^−1^ (FiO_2_ = 0.6, air/oxygen = 1:1). The partial pressure of end-tidal carbon dioxide (P_ET_CO_2_) was maintained between 35 and 45 mmHg. Continuous invasive arterial, ECG, blood pressure, oxygen saturation (SpO_2_), P_ET_CO_2_ and sevoflurane concentrations were monitored by a IntelliVue MX700 anesthesia monitor (Philips Medical Systems Boeblingen, Netherlands). Before anesthesia induction, by local anesthesia, radial artery was cannulated for invasive arterial monitor and connected to Vigileo/Flotrac system (Edwards Lifesciences, Irvine, CA, USA), and then stroke volume variation (SVV), stroke volume (SV), cardiac output (CO) and cardiac index (CI) were obtained.

### Outcome measures

The primary outcome was NLR differences perioperatively. The secondary outcomes were the postoperative complications, the incidence of postoperative ICU admission, and the length of postoperatively hospital stay. Postoperative complications include pancreatic fistula, bile leakage, chylous leakage, postoperative hemorrhage, delayed gastric emptying, and intra-abdominal infection. Major complications include pancreatic fistula Grade B / C [17], delayed gastric emptying Grade B / C [18] and severe intra-abdominal infections [19]. Other outcomes included surgery duration, pneumoperitoneum time, intraoperative fluid volume, urine volume, anesthetic consumptions (propofol, sufentanil and remifentanil) and the intraoperative vital signs (SBP, DBP, MAP, HR, CO, CI and SVV) which were recorded at four times intervals (Baseline; 0.5 h after the beginning of operation; 1 h after the beginning of operation; surgical specimen resected). Laboratory examinations were detected on preoperative (within 72 h before operation), postoperative day (POD) 0, POD1 and POD2, respectively.

### Sample size and statistical analysis

According to previous studies [20, 21], optimal cutoff values for high NLR ≥3.0, the difference between groups higher than 10% and a two-sided α level of 0.05 and power (1-*β*) to 0.8 with 80% power, we calculated that 44 patients in each group were needed. Data were analyzed using statistical product and service solutions 19.0 (SPSS Inc., Chicago, IL, USA). Results for normally distributed data for quantitative variables were expressed as mean ± SD, for non-normal distributed data were expressed as median [IQR] and for qualitative variables as percentage. Student’s *t* test was used for normally distributed data, and the Mann–Whitney *U* test was used for nonparametric data. Pearson’s χ2 test or Fisher’s exact test was used to qualitative variables. The generalized estimation equation (GEE) was allowed for analysis of repeated measurements. Repeated measure variables were expressed as estimated marginal mean (EMM) [95% confidence interval (95% CI)] and the working correlation matrix structure was independent structure and the regression model was linear regression model. *P* <0.05 was considered statistically significant.

## Results

**Fig. 1:**
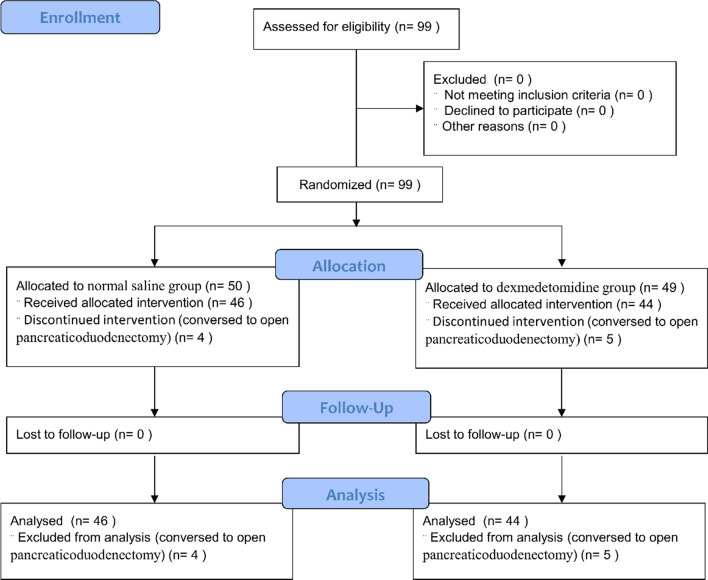
The study flow diagram of the progress

99 patients were enrolled for this trial. 9 patients were excluded because of uncontrollable hemorrhage and conversed to open pancreatoduodenectomy (OPD). 90 patients completed the study (44 in the DEX group and 46 in the NS group) (Fig. 1). Baseline patient characteristics of the trial are shown in Table 1. Preoperative variables were comparable between patients randomized for two groups. Most patients in each group were males, and the median age was 56 years. The ASA classification for all patients was grade II or III. Concomitant diseases such as hypertension and diabetes mellitus were less than 35% in both groups.

**Table 1:** Baseline patient characteristics

Variables	NS(*n* = 46)	DEX(*n* = 44)	*P*-value
Age, years, median [IQR]	55.5 [49.0 to 61.3]	56.0 [48.0 to 60.5]	0.843
BMI, kg m^−2^, mean ± SD	22.0 ± 2.5	21.7 ± 2.5	0.656
Male, *n* (%)	32 (69.6)	26 (59.1)	0.299
PTCD, *n* (%)	17 (37.0)	12 (27.3)	0.326
Premedication, n (%)	17 (37.0)	21 (47.7)	0.301
ASA class I/II/III, n	0/33/13	0/34/10	0.547
Current smoker, n (%)	21 (45.7)	17 (38.6)	0.501
Daily drinking, n (%)	14 (30.4)	10 (22.7)	0.408
Concomitant diseases, *n* (%)			
Hypertension	10 (21.7)	8 (18.2)	0.673
Diabetes mellitus	4 (8.7)	6 (13.6)	0.456
Other diseases	18 (39.1)	25 (56.8)	0.093
Preoperative vital signs			
SBP, mmHg, mean ± SD	142 ± 19	141 ± 16	0.755
DBP, mmHg, median [IQR]	72 [66 to 77]	72 [66 to 82]	0.437
MAP, mmHg, mean ± SD	95 ± 11	96 ± 12	0.557
HR, bpm, median [IQR]	79 [71 to 90]	80 [70 to 90]	0.844
CO, L min^−1^, mean ± SD	6.5 ± 1.8	6.1 ± 1.7	0.260
CI, L min^−1^ m^−2^, mean ± SD	4.1 ± 1.1	3.7 ± 1.0	0.097
SVV, %, median [IQR]	8 [6 to 10]	7 [5 to 9]	0.133

*NS*, normal saline; *DEX*, dexmedetomidine; *ASA*, American Society of Anesthesiologists;

*PTCD*, percutaneous transhepatic cholangial drainage

### Primary outcome

**Fig. 2:**
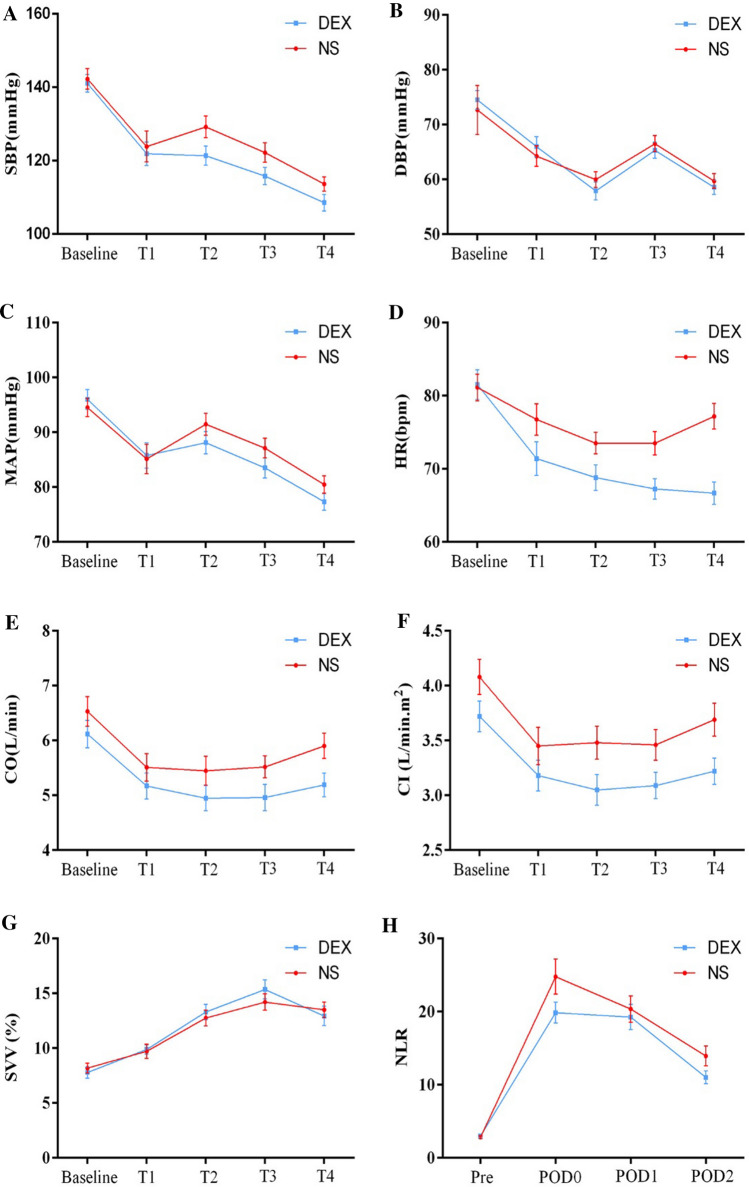
Intraoperative vital signs and NLR. Repeated measurements were analyzed by the generalized estimation equation and data are showed as EMM ± SEM. *EMM*, estimated marginal mean; *NS*, normal saline; DEX, dexmedetomidine; *T1*, after intubation; *T2*, 0.5 h after the beginning of operation; *T3*, 1 h after the beginning of operation; *T4*, surgical specimen resected; *Pre*, preoperative; *POD*, postoperative day

Postoperative NLR in both groups increased to the peak at POD0 and then decreased gradually. In DEX group, the EMM of postoperative NLR was overally lower than that in the NS group [NS vs. DEX, 15.48 (95% CI, 13.22 to 17.75) vs. 13.29 (95% CI, 11.68 to 14.90), *P* = 0.122], though there were no significant difference between groups. The change of NLR was expressed as ΔNLR (POD NLR to preoperative NLR ratio), compared with the NS group, ΔNLR at POD2 was significantly lower in the DEX group (NS vs. DEX, 4.33 [2.93 to 6.08] vs. 3.87 [2.36 to 4.77], *P* = 0.032) (Table 2, Fig. 2, H).

**Table 2:** Neutrophils-to-lymphocyte ratio (NLR)

Variables	NS(*n* = 46)	DEX(*n* = 44)	*P*-value
Pre, median [IQR]	2.62 [1.72 to 3.37]	2.70 [2.26 to 4.14]	0.617
POD0, median [IQR]	20.08 [14.20 to 29.82]	18.67 [11.68 to 25.93]	0.261
POD1, median [IQR)	18.30 [13.10 to 24.57]	16.19 [12.17 to 22.77]	0.473
POD2, median [IQR]	12.39 [7.90 to 16.32]	10.18 [7.20 to 14.74]	0.123
ΔNLR, median [IQR]			
POD0	8.39 [4.65 to 15.08]	5.94 [4.19 to 10.07]	0.125
POD1	6.40 [4.92 to 10.40]	6.03 [4.17 to 8.02]	0.366
POD2	4.33 [2.93 to 6.08]	3.87 [2.36 to 4.77]	0.032
NLR, EMM (95% CI)	15.48 (13.21 to 17.75)	13.29 (11.68 to 14.90)	0.122

*NS*, normal saline; *DEX*, dexmedetomidine; *POD*, postoperative day; Pre, preoperative; *ΔNLR*, POD NLR to Pre NLR ratio; *EMM*, estimated marginal mean; *95% CI*, 95% confidence interval

### Secondary outcomes

Approximately 78% patients suffered malignant disease, there was no statistical difference between groups (*P* = 0.91). Three patients were transferred to the ICU after surgery in the NS group, while there was none in the DEX group (*P* = 0.242). The overall incidence of major postoperative complications was 54.3% in the NS group and 38.6% in the DEX group; less major complications were observed in the DEX group such as pancreatic fistula, delayed gastric emptying and intra-abdominal infection (NS vs. DEX, 21.7% vs. 13.6%, *P* = 0.315; 10.9% vs. 2.3%, *P* = 0.226; 17.4% vs. 11.4%, *P* = 0.416, respectively) though there were no statistical differences (Table 3).

**Table 3:** Postoperative outcomes

Variables	NS(*n* = 46)	DEX(*n* = 44)	*P*-value
Malignant, *n* (%)	36 (78.3)	34 (77.3)	0.910
ICU, *n* (%)	3 (6.5)	0 (0)	0.242
Postoperative complications, *n* (%)	25 (54.3)	17 (38.6)	0.135
Major complications			
Pancreatic fistula grade B/C, *n* (%)	10 (21.7)	6 (13.6)	0.315
Delayed gastric emptying grade B/C, *n* (%)	5 (10.9)	1 (2.3)	0.226
Severe intra-abdominal infection, *n* (%)	8 (17.4)	5 (11.4)	0.416
Postoperative hospital stay, median [IQR]	15 [12 to 20]	14 [12 to 18]	0.313

*NS*, normal saline; *DEX*, dexmedetomidine

### Other outcomes

Propofol and opioid consumptions were significant less in the DEX group than in the NS group, while the duration of anesthesia was similar between groups (NS vs. DEX, Propofol 1100 [738 to 1405] vs. 760 [528 to 1125]mg, *P* = 0.004; sufentanil 35 [30 to 40] vs. 30 [26 to 35]ug, *P* = 0.009; remifentanil 2.15 [1.72 to 2.80] vs. 1.95 [1.51 to 2.00]mg, *P* = 0.012). There was no significantly difference in surgery duration, pneumoperitoneum time, intraoperative fluid volume, and urine volume (Table 4).

**Table 4:** Intraoperative outcomes

Variables	NS(*n* = 46)	DEX(*n* = 44)	*P*-value
Anesthesia duration, min, median [IQR]	530 [446 to 586]	495 [450 to 575]	0.939
Surgery duration, min, median [IQR]	428 [361 to 501]	400 [344 to 450]	0.827
Pneumoperitoneum duration, min, median [IQR]	395 [318 to 448]	355 [306 to 424]	0.431
Propofol, mg, median [IQR]	1100 [738 to 1405]	760 [528 to 1125]	0.004
Sevoflurane, ml, median [IQR]	60 [50 to 80]	63 [51 to 80]	0.749
Sufentanil, ug, median [IQR]	35 [30 to 40]	30 [26 to 35]	0.009
Remifentanil, mg, median [IQR]	2.15 [1.72 to 2.80]	1.95 [1.51 to 2.00]	0.012
Cis-atracurium, mg, mean ± SD	48 ± 15	46 ± 12	0.480
Crystalloid, ml, median [IQR]	500 [500 to 1000]	500 [500 to 1000]	0.201
Colloid, ml, median [IQR]	1000 [1000 to 1500]	1000 [1000 to 1500]	0.343
Urine volume, ml, median [IQR]	375 [300 to 550]	500 [300 to 600]	0.067
Blood loss, ml, median [IQR]	100 [100 to 200]	100 [50 to 138]	0.025
RBC transfusion, *n* (%)	1 (2.2)	1 (2.3)	0.975
FFP transfusion, *n* (%)	0 (0.0)	1 (4.3)	0.489
Vasoconstrictor, *n* (%)	4 (8.7)	7 (15.9)	0.296
Atropine, *n* (%)	6 (13.0)	9 (20.5)	0.346
Intraoperative vital signs, EMM (95% CI)			
SBP, mmHg	126 (122 to 130)	122 (118 to 125)	0.097
DBP, mmHg	67 (64 to 69)	67 (64 to 69)	0.918
MAP, mmHg	88 (85 to 90)	86 (83 to 89)	0.417
HR, bpm	76 (74 to 79)	71 (68 to 74)	0.007
CO, l min^−1^	5.8 (5.4 to 6.2)	5.3 (4.9 to 5.7)	0.072
CI, l min^−1^ m^−2^	3.6 (3.4 to 3.9)	3.3 (3.0 to 3.5)	0.026
SVV, %	12 (11 to 13)	12 (11 to 13)	0.849

*NS*, normal saline; *DEX*, dexmedetomidine; *EMM*, estimated marginal mean. *RBC*, red blood cell; *FFP*, fresh frozen plasma; *95% CI*, 95% confidence interval

Compared with the NS group, HR and CI were significantly lower in the DEX group [NS vs. DEX, HR, 76 (95% CI, 74 to 79) vs. 71 (95% CI, 68 to 74) bpm, *P* = 0.007; CI, 3.6 (95% CI, 3.4 to 3.9) vs. 3.3 (95% CI, 3.0 to 3.5) l min^−1^, *P* = 0.026]; however, the administration of atropine was similar in both groups [6 (13.0%) vs. 9 (20.5%), *P* = 0.346]. Also, there were no more patients required vasoconstrictor in the DEX group [NS vs. DEX, 7 (15.9%) vs. 4 (8.7%), *P* = 0.296]. There was no significantly difference in SBP, DBP, MAP, CO and SVV between groups, though in the DEX group, intraoperative vital signs (except SVV) were slightly lower than those of NS group (Table 4, Fig. 2, A-G).

## Discussion

Surgical resection is the mainly possible cure for pancreatic cancer; however, surgery itself induces intense stress response to cause immunosuppression and excessive pro-inflammatory responses, which could promote tumor angiogenesis and increase postoperative complications [22]. The surgical stress impaired cellular immunity that promotes the proliferation of cancer cells, allowing them to escape the surveillance of the immune system and exhibit an outgrowth pattern [23]. Recent studies have indicated that inflammation markers including serum CRP and NLR are independent predictors of disease-free survival, and overall survival after pancreaticoduodenectomy [24]. Compared with OPD, patient undergoing LPD did not reduce the postoperative inflammatory response, though the length of hospital stay maybe shorter [25, 26].

As an inflammatory indicator, NLR indicates the balance between innate and adaptive immune responses and it is an excellent indicator of inflammation and stress together. Neutrophils are a part of the innate immune response, which can exert several pro-tumor activities in cancer and promote progression through different mechanisms [27]. NLR reflecting online dynamic relationship between innate (neutrophils) and adaptive cellular immune response (lymphocytes) can sensitively represent the inflammatory level and predict the cancer prognosis in many kinds of pathological states Therefore, the higher level of NLR was related to the worse outcomes of tumor diseases^6^. Walsh et al. were the first to apply the parameter for the prognosis of cancer patients undergoing colorectal surgery [28]. The relation between inflammation and prognosis of cancer expressed by NLR has enhanced in various solid tumors in the next nearly fifty years [29−31]. Our results showed that the application of DEX in patients undergoing LPD demonstrates lower changes in NLR postoperatively and levels of NLR at all postoperative time intervals were lower, which similar to the former researches [32]. It is well known that reasonable anesthetic strategy is of importance for the outcomes of patients. Anesthetics are not only release the pain, but also play a critical role in immunomodulatory and tumor metastasis [33]. Different anesthetics play a positive or negative role in host’s immunity, which together maintain an immune balance [34]. DEX is widely used in the perioperative period and plays a beneficial role in resection of a variety of solid cancers [35]. A recent animal experiment confirmed its anti-inflammatory effects and showed a lower tumor burden [36].Clinical researches also shown that application of DEX could reduce serum levels of inflammatory factors in patients receiving carcinoma resection including colon cancer [37], hysterectomy [38], and radical gastrectomy [39]. In addition, immunosuppression attenuated by DEX could also concerned with better outcomes of postoperative cognitive function [40], effective perioperative analgesia, less use of opioids [41]. Contrary to DEX, opioids are recognized as suppressing immunity and promoting cancer proliferation, result in potential narcotic dependence, respiratory depression, and gastrointestinal dysfunctions [42]. Recent reports suggested that the use of narcotics for perioperative analgesia can cause Oddi sphincter contraction, which maybe increase intrapancreatic pressure and lead to postoperative pancreatic fistula (POPF) [43]. We found that intraoperative DEX dramatically decreased the consumption of propofol and narcotics, since the POPF often lead to catastrophic consequences [44], the minimize of opioids by DEX gave the strong excuse for exquisite further study. Intra-abdominal infection with acute physiology and chronic health evaluation II (APACHE II) ≥10, sepsis, or acute gastrointestinal injury (AGI) grade ≥ III was defined as severe intra-abdominal infection [45]. Among mechanically ventilated patients with sepsis, sedation with DEX resulted in reduced inflammatory response and increased lactate clearance [46, 47]. Intraoperative DEX reduced the time to first flatus, first oral feeding, and first defecation [48].These results showed that this treatment may be a feasible strategy for improving postoperative gastrointestinal function recovery in patients undergoing laparoscopic operation. With agreement, our results showed that the incidences of major postoperative complications including POPF, delayed gastric emptying and severe intra-abdominal infection were reduced in the DEX group though there were no statistical significance, which means further research is urgently needed.

## Limitations

There are several limitations in this study. Firstly, the inflammatory indicators we examined were relatively single, and there may be differences in other immune cells and cytokines. Furthermore, long-term indicators are important for cancer prognosis, and we only collect short-term outcome indicators and pay more attention to the immune-related situation, did not follow up after discharge from the hospital. Secondly, patients undergoing LPD include different types of periampullary carcinomas, and the bias caused by cancer type cannot be completely ruled out, although the distribution of tumor types in two groups was similar. Thirdly, we only collected and measured samples every 24 h after surgery. For the lack of detection within 24 h after surgery, the dynamic changes of inflammatory factors and immune cells may not be observed in time.

## Conclusions

In summary, the use of DEX in LPD patients may attenuate the early postoperative inflammatory response and be associated with reducing the consumption of propofol and opioids. Less major postoperative complications such as pancreatic fistula, delayed gastric emptying and intra-abdominal infection were observed in the DEX group though there were no statistical differences, additional research is urgently needed to clarify the proper anesthetic strategy for LPD.
